# Induction of HO-1 by Mevastatin Mediated via a Nox/ROS-Dependent c-Src/PDGFRα/PI3K/Akt/Nrf2/ARE Cascade Suppresses TNF-α-Induced Lung Inflammation

**DOI:** 10.3390/jcm9010226

**Published:** 2020-01-15

**Authors:** Chih-Chung Lin, Wei-Ning Lin, Rou-Ling Cho, Chien-Chung Yang, Yi-Cheng Yeh, Li-Der Hsiao, Hui-Ching Tseng, Chuen-Mao Yang

**Affiliations:** 1 Department of Anesthetics, Chang Gung Memorial Hospital at Linkuo, Kwei-San, Tao-Yuan 33302, Taiwan; chihchung@adm.cgmh.org.tw; 2 Graduate Institution of Biomedical and Pharmaceutical Science, College of Medicine, Fu Jen Catholic University, New Taipei City 24205, Taiwan; 081551@mail.fju.edu.tw; 3 Department of Pharmacology, College of Medicine, China Medical University, Taichung 40402, Taiwan; royeariel760918@gmail.com (R.-L.C.); xmasmilk@hotmail.com (Y.-C.Y.); lidesiao@livemail.tw (L.-D.H.); huiching1205@yahoo.com.tw (H.-C.T.); 4 Department of Traditional Chinese Medicine, Chang Gung Memorial Hospital at Tao-Yuan, Kwei-San, Tao-Yuan 33302, Taiwan; r55161@cgmh.org.tw; 5 School of Traditional Chinese Medicine, College of Medicine, Chang Gung University, Kwei-San, Tao-Yuan 33302, Taiwan; 6 Department of Post-Baccalaureate Veterinary Medicine, College of Medical and Health Science, Asia University, Wufeng, Taichung 41354, Taiwan

**Keywords:** mevastatin, ROS, Nrf2, AREs, heme oxygenase-1

## Abstract

Background: Mevastatin (MVS), a 3-hydroxy-3-methylglutaryl coenzyme, a reductase (HMG-CoA) inhibitor, has anti-inflammatory effects potentially via up-regulation of heme oxygenase-1 (HO-1). However, the mechanisms underlying MVS-induced HO-1 expression remain largely unknown in human pulmonary alveolar epithelial cells (HPAEpiCs). Methods: HO-1 and intercellular adhesion molecule (ICAM)-1 expression were determined using real-time PCR, Western blotting, and promoter reporter analyses. The signaling components were investigated using pharmacological inhibitors or specific small interfering RNA (siRNA)s. Interaction between Nrf2 and the antioxidant response element (ARE) binding site for the HO-1 promoter was determined by chromatin immunoprecipitation (ChIP) assay. Results: Upregulation of HO-1 by MVS attenuated the tumor necrosis factor (TNF)-α-stimulated ICAM-1 expression associated with THP-1 adhesion to HPAEpiCs. These inhibitory effects of HO-1 were reversed by tin protoporphyrin (SnPP)IX or by transfection with HO-1 siRNA. MVS-induced HO-1 expression was mediated via NADPH oxidase (Nox)-derived reactive oxygen species (ROS) generation. Activation of Nox2/ROS further stimulated the phosphorylation of p47*^phox^*, proto-oncogene tyrosine-protein kinase (c-Src), platelet-derived growth factor receptor (PDFGR)α, protein kinase B (Akt), and Nrf2, which were inhibited by siRNAs. Pretreatment with pharmacological inhibitors, including diphenyleneiodonium (DPI), apocynin (APO), N-acetyl-L-cysteine (NAC), PP1, AG1296, or LY294002, reduced the MVS-activated Nrf2 nuclear-translocation binding to the ARE on the HO-1 promoter. Conclusions: MVS-induced HO-1 is, at least in part, mediated through a p47*^phox^*/Nox2/ROS-dependent activation of c-Src/PDGFRα/PI3K/Akt-regulated Nrf2/ARE axis and suppresses the TNF-α-mediated inflammatory responses in HPAEpiCs.

## 1. Introduction

Inhalation or other exposure to risk factors in the environment might stimulate the generation of reactive oxygen species (ROS) associated with lung inflammatory diseases. Inflammation plays an important role in the development of pulmonary diseases [1]. During inflammation, leukocytes release pro-inflammatory cytokines which contact with other cells to trigger the inflammatory responses [2]. For example, tumor necrosis factor α (TNF-α) has been implicated in post-transcriptional and/or translational upregulation of adhesion molecules and inflammatory genes in respiratory diseases [3,4]. Therefore, the treatment strategy for TNF-α-induced inflammatory responses protects against the cellular immune system in pulmonary diseases [5]. Currently, no effective therapy is available for the treatment of lung diseases. Therefore, development of new drugs or repurposing old drugs that induce the expression of anti-inflammatory or antioxidant enzymes might be beneficial for protecting against lung inflammation.

Statins are inhibitors of 3-hydroxy-3-methylglutaryl-coenzyme A (HMG-CoA) reductase (HMGR), are used for the treatment of hyperlipidemia in clinics, and exert anti-inflammatory effects in various diseases [6,7]. Among them, mevastatin (MVS) acts as a protective agent that significantly reduces several pro-inflammatory cytokines-regulated inflammatory responses and oxidative stress [8,9]. MVS also ameliorates the sphingosine 1-phosphate (S1P)-stimulated cyclooxygenase-2 (COX-2)/prostaglandin E_2_ (PGE_2_)-dependent cell migration for prevention of airway inflammation [10]. MVS induces anti-oxidant enzyme HO-1 expression, which may play a role in innate defenses of the respiratory system against oxidative or inflammatory lung diseases [11,12,13]. 

Heme oxygenase (HO) is the enzyme that catalyzes the rate-limiting step in the oxidative degradation of heme into iron (Fe^2+^), carbon monoxide (CO), bilirubin-IXα (BR), and biliverdin-IXα (BV), which mediate the anti-oxidative or anti-inflammatory activities [11]. The three major isozymes of HO include HO-1 (*HMOX1*), HO-2 (*HMOX2*), and HO-3 (*HMOX3*, unclear). In mammals, HO-1 expression is low under physiological conditions, but is induced by oxidative stresses, whereas HO-2 is constitutively expressed in various tissues [14]. Upregulation of HO-1 plays a crucial role in protecting lung tissues against disruption by various insults that may result in inflammatory diseases [15,16]. HO-1 (*HMOX1^-/-^*) knockout mice or cell cultures are more susceptible to oxidative stresses, leading to tissue injury [17]. Taken together, these studies strongly suggest that HO-1 plays a major protective role in different inflammatory diseases and maintains the homeostasis of physiological functions.

NADPH oxidases (Nox) are the intercellular ROS generating membrane-bound multi-component enzyme complexes that actively communicate during the host responses to a variety of stimuli [18]. Upon activation, the cytosolic regulatory subunits (p40*^phox^*, p47*^phox^*, p67*^phox^*, Ras-related C3 botulinum toxin substrate (Rac)1, and Rac2) translocate to the membrane and assemble with membrane-bound components (gp91*^phox^*/p22*^phox^*), leading to ROS (O_2_^∙^^–^/H_2_O_2_) generation [19]. To avoid injury due to oxidative stress, cells have evolved strategies to activate cytoprotective signaling pathways, such as Nrf2/HO-1, to overcome ROS challenge [20]. Therefore, low levels of ROS might act as secondary signals to induce the expression of anti-oxidant enzymes that protect against the oxidative responses [21]. Expression of HO-1 can be induced by various insults that cause oxidative stress-related damage to the organs [22]. Although oxidative stress is implicated as a causative factor in respiratory diseases, the signaling pathways linking to HO-1 expression via Nox and ROS production have not been well characterized.

Src family kinases (SFKs) are a class of non-receptor protein tyrosine kinases that mediate several intracellular signaling pathways related to extracellular stimulations, thereby regulating a wide spectrum of cellular processes [23]. Activation of Src/signal transducer and activator of transcription (STAT)3-dependent HO-1 induction mediates chemoresistance of breast cancer cells to doxorubicin by promoting autophagy [24]. Our previous study demonstrated that Nox/ROS can modulate downstream signal components of c-Src [19]. Another group of receptor tyrosine kinases (RTKs), called cell-surface RTKs, mediates ligand-specific binding responses to intracellular tyrosine residues and transduction of signaling molecules to the downstream molecules [25]. Activation of c-Src triggers platelet-derived growth factor receptor (PDGFR)-dependent progressions of the cell cycle [26]. PDGFR-mediated HO-1 expression can protect against tissue injury to oxidative stress [27]. ROS have been shown to regulate cellular functions by activating phosphoinositide-3 kinase (PI3K)/Akt signaling pathways [28]. Simvastatin-dependent HO-1 protein induction is mediated through activation of PI3K/Akt [29]. To construct a correlation between MVS and HO-1, therefore, we conducted experiments to investigate whether c-Src/PDGFRα/PI3K/Akt is involved in the MVS-induced HO-1 expression in HPAEpiCs.

The molecular regulation of HO-1 by diverse stimuli mainly occurs at the transcriptional level, such as through nuclear factor erythroid 2-related factor 2 (Nrf2) [30,31]. The redox-sensitive transcription factor Nrf2-regulated phase II enzyme HO-1 signaling pathway has become a therapeutic target of many anti-oxidants [21]. Exposure of the cells to oxidative stress leads to inhibition of Kelch-like ECH-associated protein 1 (Keap1)-dependent ubiquitination of Nrf2, the dissociation of the Keap-1/Nrf2 complex, and the nuclear translocation of Nrf2 further binds to antioxidant response element (ARE) sequences [28,32]. Thus, we investigated whether MVS-induced HO-1 is, at least in part, mediated through Nox/ROS/c-Src/PDGFRα/PI3K/Akt-mediated Nrf2/ARE activation pathway and protects against the TNF-α-regulated pulmonary inflammatory responses.

## 2. Materials and Methods

### 2.1. Reagents and Antibodies

Dulbecco’s modified Eagle’s medium (DMEM)/F-12, fetal bovine serum (FBS), and TRIzol reagent were purchased from Invitrogen (Carlsbad, CA, USA). GenMute™ siRNA Transfection Reagent was obtained from SignaGen Laboratories (Rockville, MD, USA). Dihydroethidium (DHE), 6-chloromethyl-2′,7′-dichlorodihydrofluorescein diacetate, acetyl ester acetic 2-[3,6-bis(acetyloxy)-2,7-dichloro-9H-xanthen-9-yl]-4-(chloromethyl)benzoic anhydride (CM-H_2_DCF-DA), and (2′-7′-Bis-(2-carboxyethyl)-5-(and-6)-carboxyfluorescein/acetoxymethyl ester) (BCECCF/AM) were purchased from Molecular Probes (Eugene, OR, USA). PP1, apocynin (APO), diphenylene iodonium chloride (DPI), N-acetyl cysteine (NAC), and LY294002 were purchased from Biomol (Plymouth Meeting, PA, USA). Mevastatin (MVS) was sourced from Cayman Chemical (Ann Arbor, MI, USA). Anti-HO-1 polyclonal antibody (ADI-SPA-895) was purchased from Enzo Life Sciences (Farmingdale, NY, USA). Anti-ICAM-1 (H-108; sc-7891), anti-β-actin (C4; sc-47778), anti-c-Src (SRC 2; sc-18), anti-Akt1/2/3 (H-136) (sc-8312), anti-PDGFRα (C-20; sc-338), anti-Lamin A (H-102; sc-20680), and anti-GαS (K-20; sc-823) were purchased from Santa Cruz Biotechnology (Dallas, TX, USA). Anti-glyceraldehyde 3-phosphate dehydrogenase (GAPDH) [MCA-1D4] antibody was obtained from EnCor (Gainesville, FL, USA). Anti-intercellular adhesion molecule (ICAM)-1 (ab179707) for immunohistochemistry (IHC) staining, anti-Nox2/gp91*^phox^* (ab129068), and anti-Nrf2 (Ser^40^; ab76026) were purchased from Abcam (Cambridge, U.K.). Anti-p47*^phox^* (R12-3284) and anti-p47*^phox^* (phospho-Ser^370^; A1171) were sourced from Assay Biotech (Sunnyvale, CA, USA). Anti-phospho-c-Src family (Tyr^416^; 2101), anti-phospho-Akt (Ser^473^; 9271), and anti-phospho-PDGFRα (Tyr^1018^; 4547) were purchased from Cell Signaling (Danvers, MA, USA).

### 2.2. Animal Care and Experimental Procedures

Male Institute of Cancer Research (ICR) mice (6–8 weeks old) were purchased from the National Laboratory Animal Centre (Taipei, Taiwan) and handled according to the guidelines of Animal Care Committee of Chang Gung University (Approval Document No. CGU 16-046) and National Institutes of Health (NIH) Guides for the Care and Use of Laboratory Animals (NIH Publication No. 85-23, revised 1996). ICR mice were anesthetized and individually placed on a board in a near-vertical position and the tongues were withdrawn with lined forceps. TNF-α (0.25 mg/kg body weight) was placed posterior in the throat and aspirated into the lungs. Control mice were administrated with sterile 0.1% bovine serum albumin (BSA). Mice regained consciousness after 15 min. Mice were administered a dose of MVS (0.1 mg/kg body weight) for 24 h before TNF-α treatment, and sacrificed after 24 h. Bronchoalveolar lavage (BAL) fluid was collected through a tracheal cannula using 1 mL aliquots of ice-cold PBS solution. Leukocyte count was determined by a Z1 Coulter Counter (Beckman Coulter, Indianapolis, IN, USA) as previously described [33].

### 2.3. Immunohistochemical (IHC) Staining

IHC staining was performed on the sections of the lung tissues, which were deparaffinized, rehydrated, and washed with Tween-Tris buffered saline (TTBS). Non-specific binding was blocked by preincubation with PBS containing 5 mg/mL of BSA for 1 h at room temperature. The sections were incubated with an anti-ICAM-1 or anti-HO-1 antibody (1:100 dilution) at 4 °C for 16 h and then with anti-mouse or anti-rabbit horseradish peroxidase (HRP) antibody at room temperature for 1 h. Binding antibodies were detected by incubation in 0.5 mg/mL of 3,3-diaminobenzidine (DAB)/0.01% (v/v) hydrogen peroxide in 0.1 M Tris-HCl buffer, as a chromogen (Vector Lab, Burlingame, CA, USA). 

### 2.4. Cell Culture

HPAEpiCs were purchased from ScienCell Research Laboratories (San Diego, CA, USA). When the cultures reached 80% confluence (3 days), cells were treated with 0.05% (w/v) trypsin/1 mM ethylenediaminetetraacetic acid (EDTA) for 5 min at 37 °C. The cell suspensions were plated onto 6-well culture plates at 2 mL/well and 10cm culture dishes at 10 mL/dish for the measurement of protein expression and messenger RNA (mRNA) accumulation, respectively. Four to six passages of HPAEpiCs were used throughout this study. Cells were incubated with 0.5% dimethyl sulfoxide (DMSO) (control), or MVS (3, 10, and 30 μM) for 24 h, and cell viability was determined using a 2,3-bis-(2-methoxy-4-nitro-5-sulfophenyl)-2H-tetrazolium-5-carboxanilide (XTT) assay kit according to the manufacturer’s instructions (Sigma Aldrich, St. Louis, MO, USA).

### 2.5. Protein Preparation and Western Blot Analysis

Growth-arrested HPAEpiCs were incubated with or without different concentrations of MVS at 37 °C for the indicated time intervals. When inhibitors were used, they were added 1 h prior to the application of MVS. After incubation, the cells were then rapidly washed with ice-cold PBS and lysed with 1.25 × sample buffer. After collection, samples were heated for 12 min at 95 °C. The mixed samples (15 μL) were subjected to sodium dodecyl sulfate-polyacrylamide gel electrophoresis (SDS-PAGE) using a 10% running gel. Proteins were transferred to nitrocellulose (NC) membranes, incubated with a primary antibody at 4 °C overnight, and then washed with TTBS several times and incubated with a 1:2000 dilution of an anti-rabbit or anti-mouse antibody for 1 h. Following incubation, the membranes were extensively washed with TTBS. The immunoreactive bands were detected by enhanced chemiluminescence (ECL) reagents and captured by a UVP BioSpectrum 500 Imaging System (Upland, CA, USA). The image densitometry analysis was conducted using UN-SCAN-IT gel software (Orem, UT, USA), as described previously [33].

### 2.6. Total RNA Extraction and Real-Time Quantitative (q)PCR Analysis 

Total RNA was isolated from HPAEpiCs treated with MVS for the indicated time intervals in 10 cm culture dishes with TRIzol according to the manufacturer’s instructions. mRNA was reverse-transcribed into cDNA and analyzed by real-time quantitative PCR (RT-qPCR). RT-qPCR was performed with a StepOnePlus^TM^ real-Time PCR System (ThermoScientific-Applied Biosystems, San Mateo, CA, USA) and Kapa Probe Fast qPCR Kit Master Mix (2×) Universal (KK4705; KAPA Biosystems, Wilmington, MA, USA). The levels of HO-1 and ICAM-1 expressions were quantified by normalization to GAPDH expression. Relative gene expression was determined using the ΔΔ^Ct^ method, where Ct = threshold cycle. All experiments were performed in triplicate.

### 2.7. Adhesion Assay

HPAEpiCs were plated on 6-well culture plates with coverslips and pretreated with MVS for 1 h, and then incubated with or without TNF-α for 16 h at 37 °C in a humidified 5% CO_2_ atmosphere. THP-1 cells (human monocytic cell line, BCRC 60430, RRID:CVCL_0006) were purchased from Bioresource Collection and Research Center (Hsinchu, Taiwan). BCECF-AM (10 µM) was added to THP-1 in warm PBS for 1 h at 37 °C. After labeling, cells were washed three times and re-suspended in warm PBS and maintained in the dark at room temperature. Then, the labeled THP-1 cells were added to HPAEpiCs. Non-adherent cells were removed from the plate by gentle washing with warm PBS. The numbers of adherent THP-1 cells were determined by counting four fields per 200 × high-power field well using a fluorescence microscope (Zeiss, Axiovert 200M, Baden-Württemberg, Germany). 

### 2.8. Transient Transfection with siRNAs in HPAEpiCs

SMARTpool RNA duplexes corresponding to p47*^phox^* (SASI_Hs02_00302212), Nox2 (SASI_Hs01_00086110), c-Src (SASI_Hs01_00112905), Akt (SASI_Hs01_00105954), Nrf2 (SASI_Hs02_00302212), and Scrambled control siRNA were obtained from Sigma Aldrich (St. Louis, MO, USA). PDGFRα (HSS107751, HSS182125, HSS182126) was purchased from Invitrogen Life Technologies (Carlsbad, CA, USA). Transient transfection of siRNAs was achieved by using Opti-MEM and GenMute reagent. The transfection complex (siRNA 100 nM, Opti-MEM 100 μL, and Genmute reagent 2.5 μL) was directly added to the cells and incubated for 5 h. The cells were replaced with DMEM/F-12 medium containing 10% FBS and left overnight and then changed to serum-free medium for 48 h. 

### 2.9. Immunofluorescence (IF) Staining

HPAEpiCs were plated on 6-well culture plates with coverslips. After treatment with MVS, cells were washed twice with ice-cold PBS, the cells were fixed with 4% paraformaldehyde in PBS for 20 min, and then permeabilized with 0.3% Triton X-100 in PBS for 15 min. The staining was performed by incubating with 10% normal goat serum in PBS for 30 min, followed by incubating with an anti-Nrf2 antibody (1:200 dilution) in PBS with 1% BSA and left overnight, washing three times with PBS. Then, incubation for 1 h followed with a fluorescein isothiocyanate (FITC)-conjugated goat anti-rabbit antibody (1:200 dilution) in PBS with 1% BSA, washing three time with PBS, and finally mounting with aqueous mounting medium. The images were observed under a fluorescence microscope (Zeiss, Axiovert 200M, Baden-Württemberg, Germany).

### 2.10. NADPH Oxidase Activity Assay

The NADPH oxidase inhibitor or other signal transduction inhibitors were pretreated for 1 h and then stimulated by 30 μM MVS for the indicated time intervals. After exposure to 30 μM MVS, cells were gently scraped and centrifuged at 16,000× *g* for 10 min at 4 °C. The cell pellets were re-suspended in a known volume (35 μL/per well) of ice-cold PBS, and the cell suspensions were kept on ice. Neither NADPH nor NADH enhanced the background chemiluminescence of lucigenin alone (30–40 counts/min). Chemiluminescence was continuously measured for 12 min, and the activity of Nox is expressed as counts/min. The calculated numbers of Nox activity were calibrated with protein concentration. The equal amount of warmed PBS medium (containing NADPH and lucigenin) was used as the blank and the untreated cells were the basal group.

### 2.11. Measurement of Intracellular ROS Accumulation 

Fluorescent H_2_DCFDA and DHE probes were used to monitor net intracellular accumulation of ROS. HPAEpiCs were incubated with various concentrations of MVS, washed with warm PBS, and incubated in PBS or phenol red-free medium containing H_2_DCFDA (10 μM) or DHE (5 μM) at 37 °C for 30 or 10 min. Subsequently, the PBS or medium containing DCFH-DA was removed and replaced with fresh medium. The levels of fluorescence intensities (relative fluorescence units) for DCF and DHE were measured at 485/530 nm and 518/605 nm, respectively, using a fluorescence microplate reader (Synergy^H1^ Hybird Reader, BioTek, Chittenden, VT, USA) and FACSCalibur equipped with CellQuest software (BD Biosciences, Santa Clara, CA, USA). For immunofluorescence (IF) staining, after washing three times with cold-PBS, the images were observed under a fluorescence microscope (Zeiss, Axiovert 200M, Baden-Württemberg, Germany).

### 2.12. Transfection and Promoter Luciferase Assay 

For construction of the ARE-luc on human HO-1 promoter, the region spanning −3106 to 186 bp was cloned into a pGL3-basic vector (Promega, Madison, WI, USA). ARE-luc activity was determined using a luciferase assay system (Abcam, Cambridge, UK), as previously described [19]. Finally, luciferase activities were standardized to β-gal activity.

### 2.13. Isolation of Subcellular Fractions

After incubation with MVS, cells were scraped into a 1.5 mL tube. The suspension was sonicated for 10 s at output 1.5 with a sonicator (Misonix, Farmingdale, NY, USA), and centrifuged at 16,000× *g* for 10 min at 4 °C. The pellet was re-suspended in 300 μL of homogenization buffer B (pH 8.0) and sonicated for 10 s. The protein concentration of each sample was determined using the BCA reagents. Samples from these supernatant fractions (30 μg protein) were denatured, and then subjected to SDS-PAGE using a 10% (w/v) running gel and transferred to a nitrocellulose membrane. The p47*^phox^* and Nrf2 translocation levels were determined by Western blotting using anti-p47*^phox^*, anti-Gαs, anti-Nrf2, anti-Lamin A, or anti-GAPDH antibodies. The immunoreactive bands were detected by ECL reagents, as previously described [33].

### 2.14. Coimmunoprecipitation Assay

HPAEpiCs lysates containing 1 mg of protein were incubated with 2 μg of c-Src antibody at 4 °C for 1 h, and then 20 μL of 50% protein A-agarose beads was added and mixed at 4 °C for 16 h. The immunoprecipitates were collected, washed three times with immunoprecipitation (IP) lysis buffer, and then subjected to electrophoresis on 10% SDS-PAGE. Western blot analysis was performed using an anti-PDGFRα antibody.

### 2.15. Chromatin Immunoprecipitation (ChIP) Assay

After treatment with MVS (30 μM), protein-DNA complexes were fixed by 37% formaldehyde in medium. The cell lysates were sonicated at 4 °C until the DNA size was 300–500 base pairs. One portion of the samples was used as a DNA input control, and the remaining was subdivided into several portions and then incubated with or without an anti-Nrf2 antibody. The cross-linking protein-DNA complexes were reversed by incubation at 65 °C. The DNA was extracted, re-suspended in H_2_O, and then subjected to PCR amplification with the forward primer ARE. PCR products for SYBR Green primer pairs were verified to produce single products by high resolution melt curve. The mRNA levels were calculated using the ΔΔ^–^^Ct^ method. 

### 2.16. Data and Statistical Analysis

All data are expressed as the mean ± standard error of the mean (SEM). Statistical analysis was performed by using GraphPad Prizm Program 6.0 software (GraphPad, San Diego, CA, USA). We used one-way ANOVA followed by Dunnett’s post-hoc test when comparing more than two groups of data, and one-way ANOVA, nonparametric Kruskal–Wallis test, followed by Dunnett’s post hoc test when comparing multiple independent groups. *P*-values of 0.05 were considered to be statistically significant. Post tests were run only if F achieved *P* < 0.05 with no significant variance in homogeneity. Error bars were omitted when they fell within the dimensions of the symbols.

## 3. Results

### 3.1. Upregulation of HO-1 Inhibits the TNF-α-Mediated Inflammatory Response 

**Figure 1:**
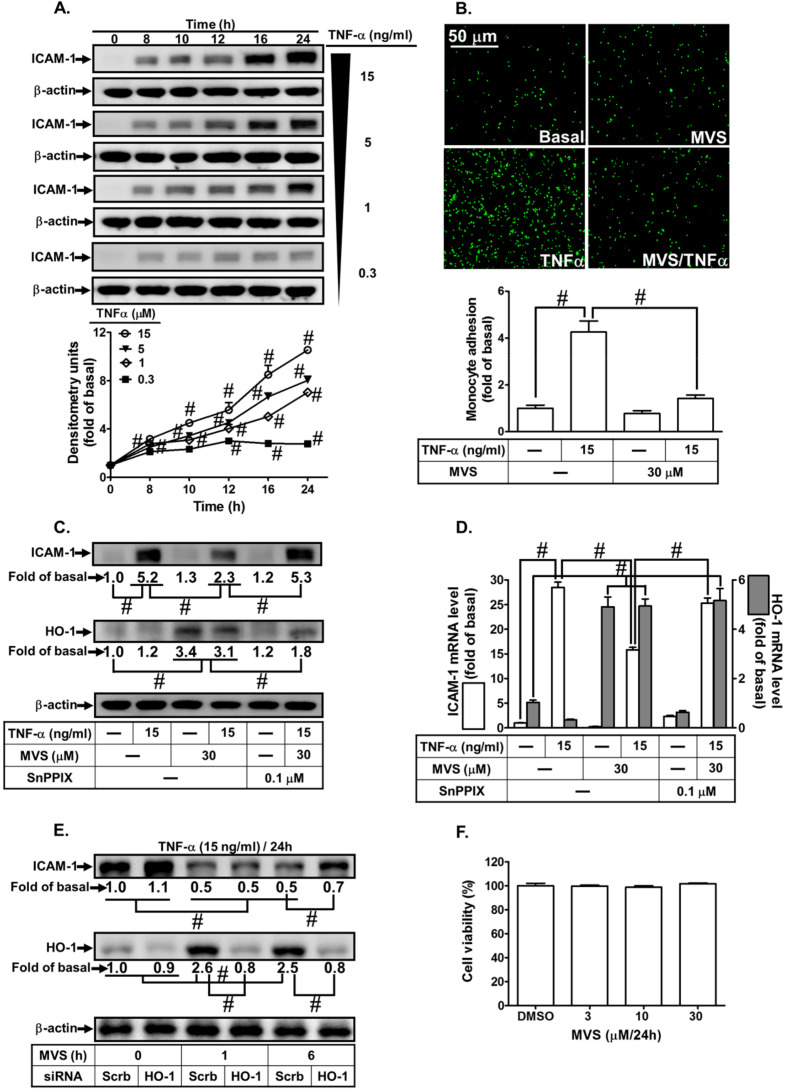
Upregulation of heme oxygenase-1 (HO-1) by mevastatin (MVS) attenuated tumor necrosis factor (TNF)-α-induced intercellular adhesion molecule (ICAM)-1 expression associated with monocyte adhesion. (**A**) human pulmonary alveolar epithelial cells (HPAEpiCs) were incubated with different concentrations of TNF-α (15, 5, 1, or 0.3 ng/mL) for the indicated time intervals. The levels of ICAM-1 and β-actin expression were determined by Western blot. (**B**–**D**) Cells were pretreated MVS for 1 h, then incubated with or without tin protoporphyrin (SnPP)IX for 1 h, and finally stimulated with TNF-α for 24 h (protein and cell adhesion) or 4 h (messenger (m)RNA). (**E**) Cells were transfected with scrambled (Scrb) or HO-1 small interfering RNA (siRNA), incubated with MVS (30 μM) for 1 or 6 h, and then incubated with TNF-α (15 ng/mL) for 24 h. (B) The adhesion of THP-1 cells (a human monocytic leukemia cell line) was measured. (**C**–**E**) The levels of ICAM-1, HO-1, and β-actin protein and mRNA were determined by Western blot and real-time PCR, respectively. (**F**) Cells were incubated with 0.5% dimethyl sulfoxide (DMSO) (control), or MVS (3, 10, and 30 μM) for 24 h and cell viability was determined using a 2,3-bis-(2-methoxy-4-nitro-5-sulfophenyl)-2H-tetrazolium-5-carboxanilide (XTT) assay kit. Data are expressed as mean ± standard error of the mean (SEM) from five independent experiments (*n* = 5). ^#^*P* < 0.01 as compared to (**A**) cells exposed to vehicle alone, or (**B**–**E**) significantly different as indicated.

**Figure 2:**
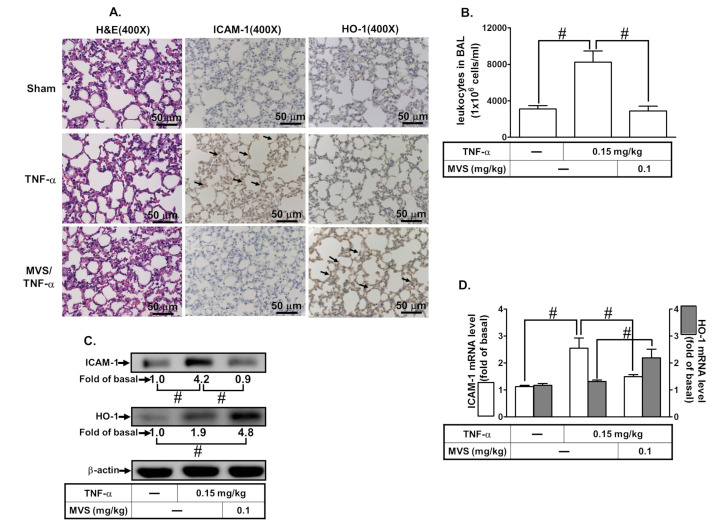
MVS alleviates TNF-α-stimulated pulmonary inflammatory responses in vivo. Institute of Cancer Research (ICR) mice were intra-peritoneal injected with MVS (0.1 mg/kg) or vehicle for 1 h, then intra-tracheally administered with or without TNF-α (0.15 mg/kg) for 24 h. (**A**) hematoxylin and eosin (H&E) and immunohistochemical staining for ICAM-1 and HO-1 in serial sections of the lung tissues from Sham (0.1 mL of DMSO-phosphate-buffered saline (PBS) (1:100) with 0.1% bovine serum albumin (BSA) treated mice), TNF-α (TNF-α-treated mice), and MVS/TNF-α (MVS plus TNF-α mice). The arrow indicates pulmonary alveolar cells expressed with ICAM-1 and HO-1. (**B**) The bronchoalveolar lavage fluid (BALF) was collected and leukocytes count was determined using a Z1 Coulter Counter. (**C**,**D**) Lung tissues were homogenized to extract protein and mRNA. The levels of ICAM-1 and HO-1 protein and mRNA were determined by Western blot and real-time PCR, respectively. Data are expressed as mean ± SEM, from five independent experiments (*n* = 5). ^#^*P* < 0.01 compared with the respective values significantly different as indicated.

Upregulation of HO-1 has been shown to reduce activation of transcription factors and production of inflammatory mediators induced by various insults [10]. Therefore, we assessed the effect of HO-1 expression by MVS on the TNF-α-induced ICAM-1 expression using in vivo and in vitro studies. First, we evaluated the effects of various concentrations of TNF-α (0.3, 1, 5, and 15 ng/mL) on the expression of ICAM-1 on HPAEpiCs. As shown in Figure 1A, TNF-α induced a time- and concentration-dependent ICAM-1 protein expression. Up-regulation of adhesion molecules is associated with the recruitment of leukocytes to the pulmonary alveolar epithelial cells [3,4]. As expected, we found that adhesion of THP-1 to HPAEpiCs challenged with TNF-α increased, which was repressed by pretreatment with MVS (Figure 1B). We investigated the effect of HO-1 induction by MVS on the TNF-α-induced responses. The cells were pretreated 30 μM MVS for one hour, continuously incubated with or without 0.1 μM SnPPIX for one hour, and then treated with 15 ng/mL TNF-α for 16 h. The levels of HO-1 protein and mRNA expression were significantly increased with MVS, which was accompanied by reduction in ICAM-1 expression challenged with TNF-α, and was reversed by co-incubation with SnPPIX (Figure 1C,D). We confirmed the inhibitory effects of HO-1 induction by MVS on the TNF-α-upregulated ICAM-1 expression by transfection with HO-1 siRNA. We found that MVS attenuated the TNF-α-induced ICAM-1 expression, which was partially reversed by transfection with HO-1 siRNA (Figure 1E). Treatment with various concentrations of MVS for 24 h produced no significant change in the cell viability of HPAEpiCs (Figure 1F). We also confirmed these results through an in vivo study. To compare the distribution of HO-1 in the murine lung tissues of TNF-α and treatment with MVS+TNF-α mice, IHC staining (Figure 2A) for ICAM-1 or HO-1 expression was determined by the DAB interaction with anti-HRP; the colorimetric detection appeared brown. ICAM-1 expression on the lung tissues of TNF-α-treated mice was attenuated by MVS-induced HO-1 expression. We found that the increase in leukocytes in the BAL fluid of mice challenged with TNF-α decreased with MVS (Figure 2B). TNF-α significantly enhanced ICAM-1 protein and mRNA expression (Figure 2C,D), which were attenuated by MVS-induced HO-1 expression in lung tissues. These results suggested that up-regulation of HO-1 by MVS protects pulmonary tissues against the TNF-α-mediated the expression of inflammatory responses. 

### 3.2. Statins Induce HO-1 Expression

**Figure 3:**
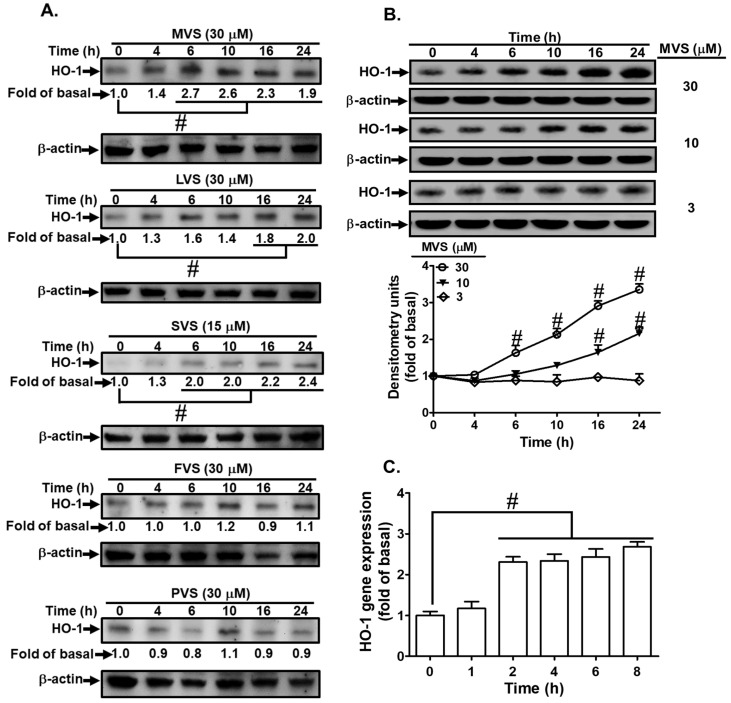
Statins induce HO-1 expression in HPAEpiCs. (**A**) The effects of statins on HO-1 expression; the cells were incubated with MVS, lovastatin (LVS), simvastatin (SVS), fluvastatin (FVS), and pravastatin (PVS) for the indicated time intervals. (**B**) HPAEpiCs were incubated with different concentrations of MVS (3, 10, and 30 μM) for the indicated time intervals. (**A**,**B**) The levels of HO-1 and β-actin protein expression were determined by Western blot. (**C**) Total RNA was isolated from HPAEpiCs treated with MVS (30 μM) for the indicated time intervals. The levels of HO-1 mRNA were determined by real-time PCR. Data are expressed as mean ± SEM from five independent experiments (*n* = 5). ^#^ *p* < 0.01 compared with the cells exposed to vehicle (0 h) alone.

Statins consists of different members; we chose MVS, lovastatin (LVS), simvastatin (SVS), fluvastatin (FVS), and pravastatin (PVS) to evaluate their potencies on HO-1 protein expression in HPAEpiCs. Cells were incubated with MVS (30 μM), LVS (30 μM), SVS (15 μM), FVS (30 μM), and PVS (30 μM) for the indicated time intervals. As shown in Figure 3A, after 24 h incubation with statins, MVS produced a significantly maximum and stable expression of HO-1 and was used for further studies. The cells were treated with various concentrations of MVS for the indicated time intervals. MVS induced HO-1 protein expression in a time- and concentration-dependent manner (Figure 3B). We observed a significant increase within 10 h, reaching a maximum response within 24 h. The levels of HO-1 mRNA were also enhanced by 30 μM MVS, determined by real-time PCR (Figure 3C). These results demonstrated that MVS-induced HO-1 expression was dependent on the concentrations of MVS used and incubation time in HPAEpiCs.

### 3.3. p47^phox^-Dependent Nox/ROS Generation Is Required for MVS-Induced HO-1 Expression

**Figure 4:**
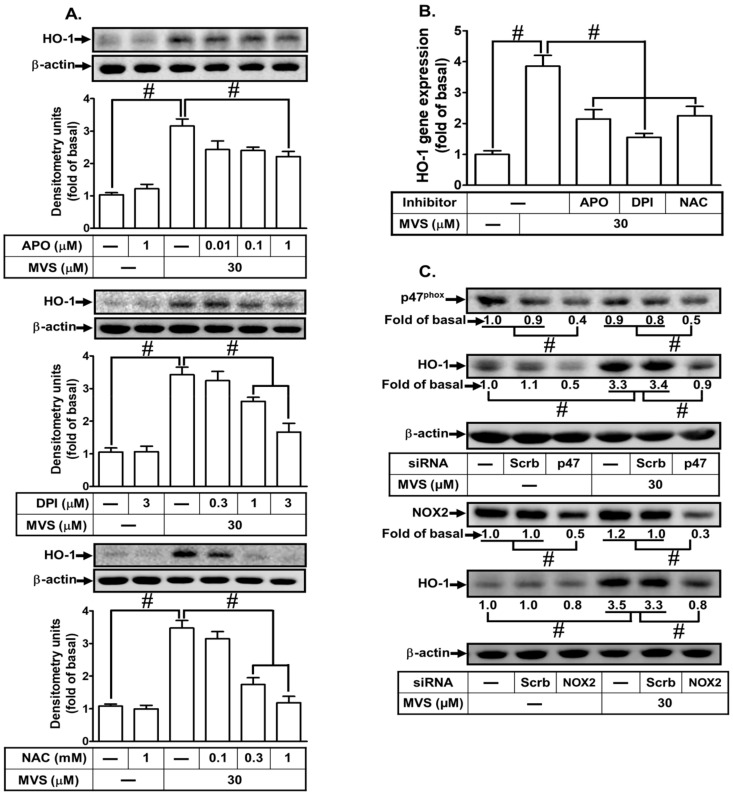
NADPH oxidase and reactive oxygen species (ROS) generation are involved in MVS-increased HO-1 expression. (**A**) Cells were pretreated with various concentrations of apocynin (APO), diphenyleneiodonium (DPI), or N-acetylcysteine (NAC) for 1 h, and then incubated with vehicle or MVS (30 μM) for 24 h. The levels of HO-1 and GAPDH protein were determined by Western blot. (**B**) The cells were pretreated with NAC (1 mM), APO (1 μM), and DPI (3 μM) for 1 h and then incubated with vehicle or MVS (30 μM) for 8 h. The levels of HO-1 mRNA were determined by real-time PCR. (**C**) HPAEpiCs were transfected with p47*^phox^* or Nox2 siRNA, and then incubated with MVS for 24 h. The levels of HO-1, p47*^phox^*, Nox2, and β-actin proteins were determined by Western blot. Data are expressed as mean ± SEM from five independent experiments (*n* = 5). ^#^*P* < 0.01 compared with the respective significantly different values as indicated.

Increase in intracellular ROS acts as a second messenger to provoke nuclear transcription factors leading to *HOMX-1* gene expression to protect against inflammatory responses [20,21]. Our previous study showed that activation of p47*^phox^/*Nox2-dependent ROS generation plays crucial roles in HO-1 expression in HPAEpiCs [19]. To examine whether p47*^phox^/*Nox/ROS mediates MVS-induced HO-1 expression in HPAEpiCs, cells were pretreated with the inhibitors of p47*^phox^* (apocynin, APO), Nox (diphenyleneiodonium, DPI), and ROS (N-acetyl-L-cysteine, NAC). Pretreatment with these inhibitors concentration-dependently attenuated the MVS-induced HO-1 expression (Figure 4A,B). To confirm the role of p47*^phox^* and Nox2 in MVS-induced responses, the protein levels of p47*^phox^* and Nox2 were knocked down by their respective siRNAs and reduced the MVS-induced HO-1 expression in HPAEpiCs (Figure 4C). 

**Figure 5:**
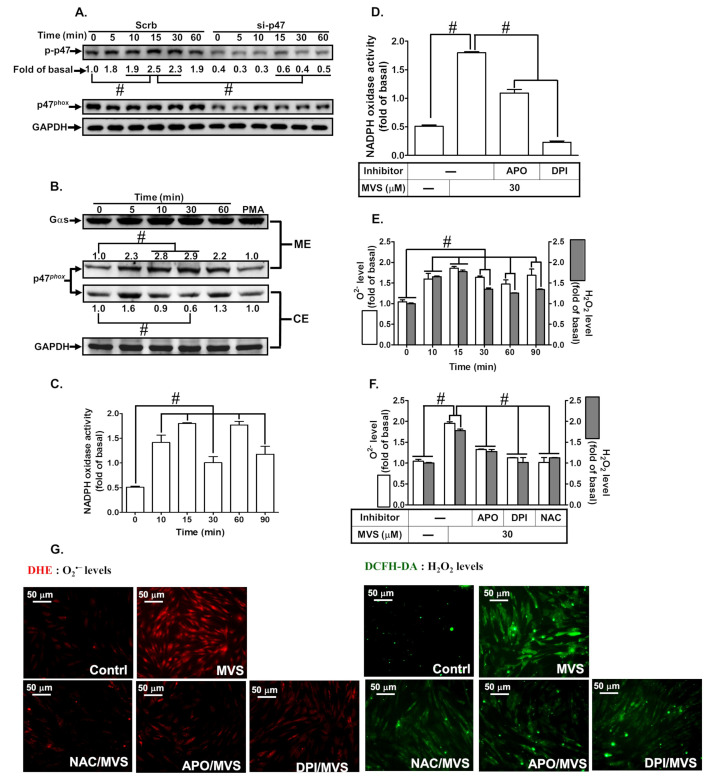
Interaction between p47*^phox^* and Nox contributes to MVS-induced HO-1 expression. (**A**) HPAEpiCs were transfected with p47*^phox^* siRNA and then incubated with vehicle or MVS (30 μM) for the indicated time intervals. The levels of p47^phox^ phosphorylation, total p47^phox^, and GAPDH were determined by Western blot using anti-phospho-p47*^phox^*, anti-p47*^phox^*, or anti-GAPDH antibody. (**B**) Cells were pretreated with MVS (30 μM) for the indicated time intervals. The cytosol and membrane fractions were prepared and analyzed by Western blot using anti-p47*^phox^*, anti-GAPDH, or anti-Gαs antibody. HPAEpiCs were treated with MVS for the indicated time intervals. The levels of (**C**,**D**) NADPH oxidase activity and (**E**–**G**) ROS generation (H_2_O_2_ or O_2_**^.–^**) were determined by ELISA or immunofluorescence (IF), reaching a maximal response within 15 min for Nox activity or 30 min for ROS. MVS stimulates Nox activity leading to ROS production, cells were pretreated with Nox or ROS inhibitors for 1 h, and then incubated with vehicle or MVS (30 μM) for (**D**) 15 min for Nox activity or (**F**,**G**) 30 min for ROS generation. MVS-induced Nox activity and ROS production were reduced by pretreatment with NAC (1 mM), APO (1 mM), and DPI (3 μM) in HPAEpiCs. Data are expressed as mean ± SEM from five independent experiments (*n* = 5). ^#^*P* < 0.01 compared with the respective significantly different values as indicated.

Previous studies have shown that phosphorylation of p47*^phox^* leading to NADPH oxidase/ROS-dependent HO-1 expression could protect against the release of inflammatory mediators from HPAEpiCs [19,34]. First, we found that MVS-stimulated phosphorylation of p47*^phox^* was reduced by transfection with p47*^phox^* siRNA (Figure 5A). To investigate the recruitment of p47*^phox^* to the membrane bound component, we found that MVS stimulated the recruitment of p47*^phox^* from the cytosolic to the membrane fraction occurring within 5 min and slightly declining at 60 min (Figure 5B). To directly determine whether MVS stimulates p47*^phox^* recruitment leading to NADPH oxidase activation, MVS time-dependently stimulated NADPH oxidase activity (Figure 5C), which was reduced by pretreatment with APO (100 μM) or DPI (1 μM) (Figure 5 D). The levels of intracellular O_2_^–^ and H_2_O_2_ were elevated by MVS as determined by DHE and DCFH-DA, respectively (Figure 5E,F). The levels of intracellular ROS accumulation reached a maximum within 10 to 15 min, which were inhibited by APO, DPI, or NAC (Figure 5F). These results were further supported by the fluorescent images observed under a fluorescence microscope (Figure 5G). These results suggested that MVS-induced HO-1 expression is mediated via p47*^phox^* phosphorylation/Nox-dependent ROS generation in HPAEpiCs. 

### 3.4. MVS Induces HO-1 Expression VIA a c-Src/PDGFRα/PI3K/Akt-Dependent Cascade

**Figure 6:**
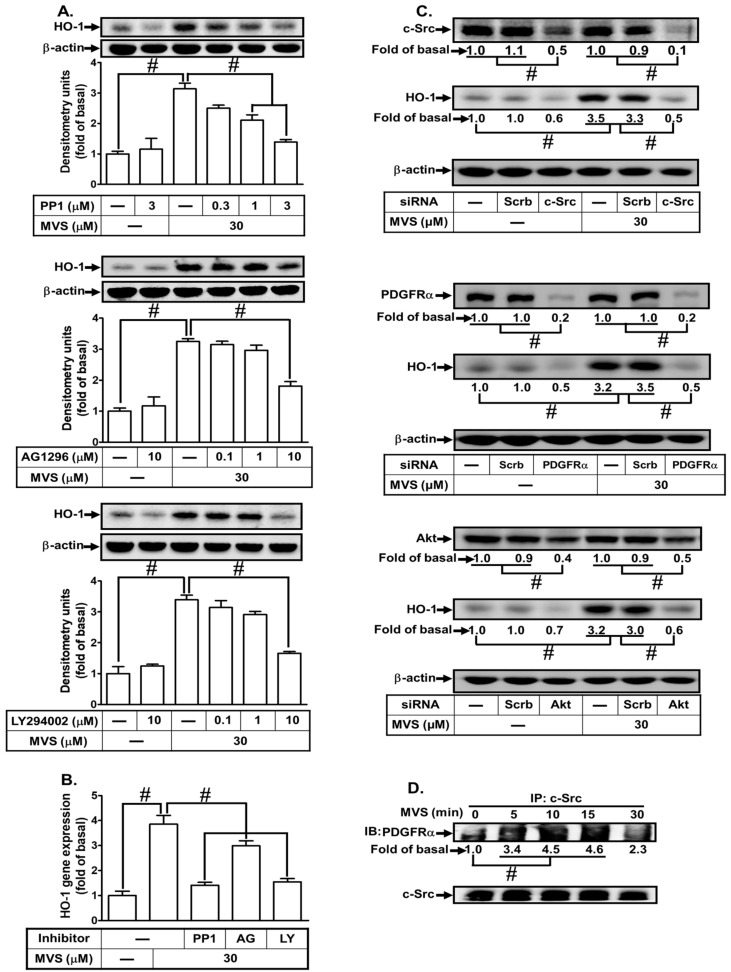
Phosphorylation of proto-oncogene tyrosine-protein kinase (c-Src)/platelet-derived growth factor receptor (PDGFR) and Akt involves in the MVS-induced HO-1 expression. (**A**) HPAEpiCs were pretreated with various concentrations of PP1 (0.3, 1, and 3 μM), AG1296 (0.1, 1, and 10 μM), or LY294002 (0.1, 1, and 10 μM) for 1 h, and then incubated with vehicle or MVS (30 μM) for 24 h. The levels of HO-1 and β-actin protein expression were determined by Western blot using an anti-HO-1 or anti-β-actin antibody. (**B**) The cells were pretreated with PP1 (3 μM), AG1296 (10 μM), or LY294002 (10 μM) for 1 h and then incubated with vehicle or MVS (30 μM) for 8 h. The levels of HO-1 mRNA were analyzed by real-time PCR. (**C**) HPAEpiCs were transfected with Scrb, c-Src, PDGFRα, or Akt siRNA, and then incubated with MVS (30 μM) for 24 h. The levels of c-Src, PDGFRα, Akt, HO-1, and β-actin protein expression were determined by Western blot using anti-c-Src, anti-PDGFRα, anti-Akt, anti-HO-1, or anti-GAPDH antibody. (**D**) Cells were treated with MVS (30 μM) for the indicated time intervals and subjected to immunoprecipitation using an anti-c-Src antibody. The immunoprecipitates were analyzed by Western blot using the antibody as indicated. Data are expressed as mean ± SEM from five independent experiments (*n* = 5). ^#^*P* < 0.01 compared with the respective significantly different values as indicated.

In our previous studies, Src family kinases (SFKs) were found to play a role in inducing the expression of anti-oxidant enzymes, such as HO-1, to exert anti-inflammatory effects in the respiratory system [19,35]. PDGFRα and PI3K/Akt are downstream components of Src that regulate HO-1 expression in various cell types [36]. To determine whether MVS-induced HO-1 expression is mediated through activation of the ROS-dependent c-Src/PDGFRα/PI3K/Akt cascade in HPAEpiCs, the inhibitors of c-Src (PP1), PDGFRα (AG1296), and PI3K/Akt (LY294002) were used. Pretreatment with PP1, AG1296, or LY294002 significantly attenuated MVS-induced HO-1 protein and mRNA (Figure 6A,B). Verifying the roles of c-Src, PDGFRα, or Akt in the MVS-induced HO-1 expression, transfection with c-Src, PDGFRα or Akt siRNA down-regulated the levels of their respective proteins and also inhibited the MVS-induced HO-1 expression (Figure 6C). ROS/Src have been shown to activate PDGFRα and thereby promote proliferation and survival of cells [26]. Thus, we further investigated the physical interaction between c-Src and PDGFRα via immunoprecipitation using an anti-c-Src antibody. As shown in Figure 6D, MVS induced the formation of c-Src/PDGFRα complex reaching a maximum within 10–15 min in these cells. 

**Figure 7:**
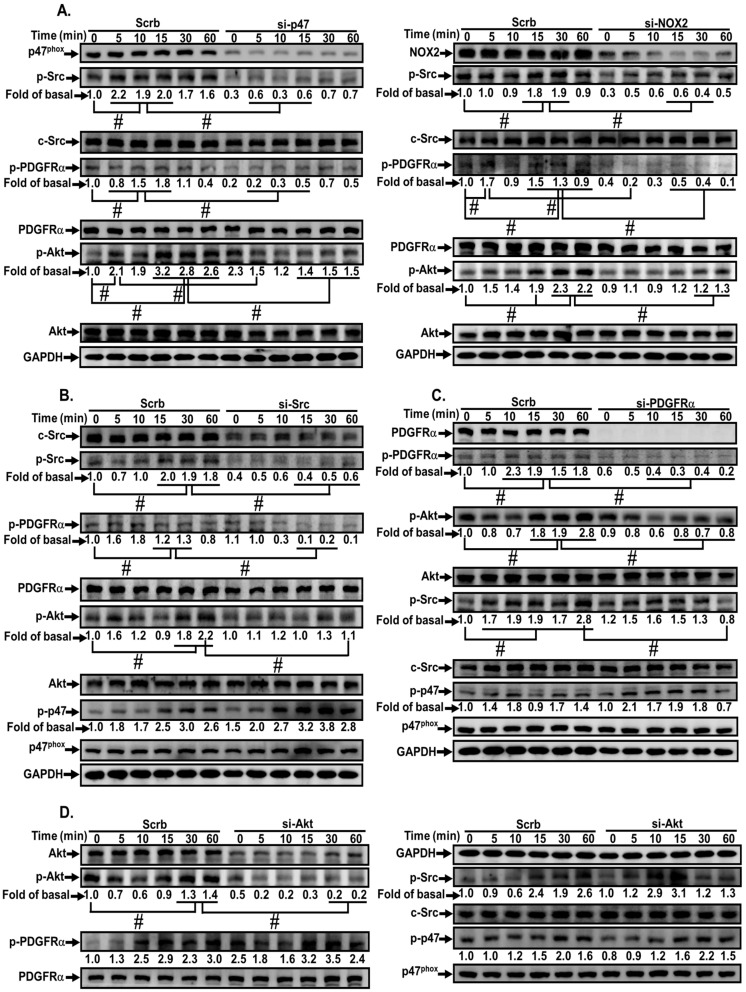
MVS-induced HO-1 expression via a p47*^phox^*/NOX/ROS-dependent c-Src/PDGFRα/PI3K/Akt pathway. HPAEpiCs were (**A**) transfected with p47*^phox^* (left) or Nox2 (right) siRNA, (**B**) c-Src siRNA, (**C**) PDGFRα siRNA, or (**D**) Akt siRNA, and then incubated with vehicle or MVS (30 μM) for the indicated time intervals. Western blot was performed using anti-phospho-p47, anti-p47*^phox^*, anti-phospho-c-Src, anti-c-Src, anti-phospho-PDGFRα, anti-PDGFRα, anti-phospho-Akt, anti-Akt, or anti-GAPDH antibody. Data are expressed as mean ± SEM from five independent experiments (*n* = 5). ^#^*P* < 0.01 compared with the respective significantly different values as indicated.

We also clarified the relationship among ROS-activated upstream and downstream components. MVS stimulated c-Src, PDGFRα, and Akt phosphorylation in a time-dependent manner, which was attenuated by transfection with p47*^phox^* or Nox2 siRNA during the period observation (Figure 7A), implying that c-Src, PDGFRα, and Akt are the downstream components of p47*^phox^*/Nox/ROS cascade. Transfection with c-Src siRNA also attenuated the MVS-stimulated phosphorylation of c-Src, PDGFRα, and Akt, but not p47*^phox^* phosphorylation (Figure 7B), indicating that MVS-stimulated c-Src-dependent PDGFRα and Akt phosphorylation is mediated through Nox/ROS. Transfection with PDGFRα siRNA reduced the phosphorylation of PDGFRα and Akt, but not c-Src and p47*^phox^* (Figure 7C), implying that PDGFRα and Akt are downstream components of the NOX/ROS/c-Src cascade. Transfection with Akt siRNA only attenuated Akt phosphorylation, but had no effect on phosphorylation of c-Src, PDGFRα and p47*^phox^* (Figure 7D). These results suggested that MVS-induced HO-1 expression is mediated through sequential activation of p47*^phox^*/Nox2/ROS/c-Src/PDGFRα/PI3K/Akt pathway in HPAEpiCs.

### 3.5. Roles of Nrf2/ARE in MVS-Induced HO-1 Expression 

**Figure 8:**
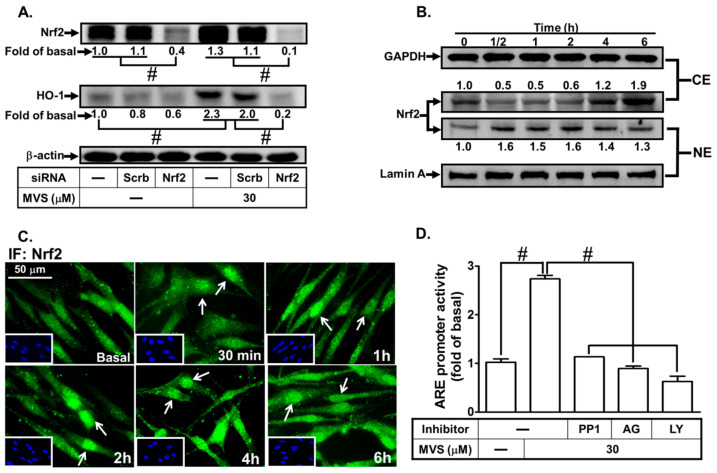

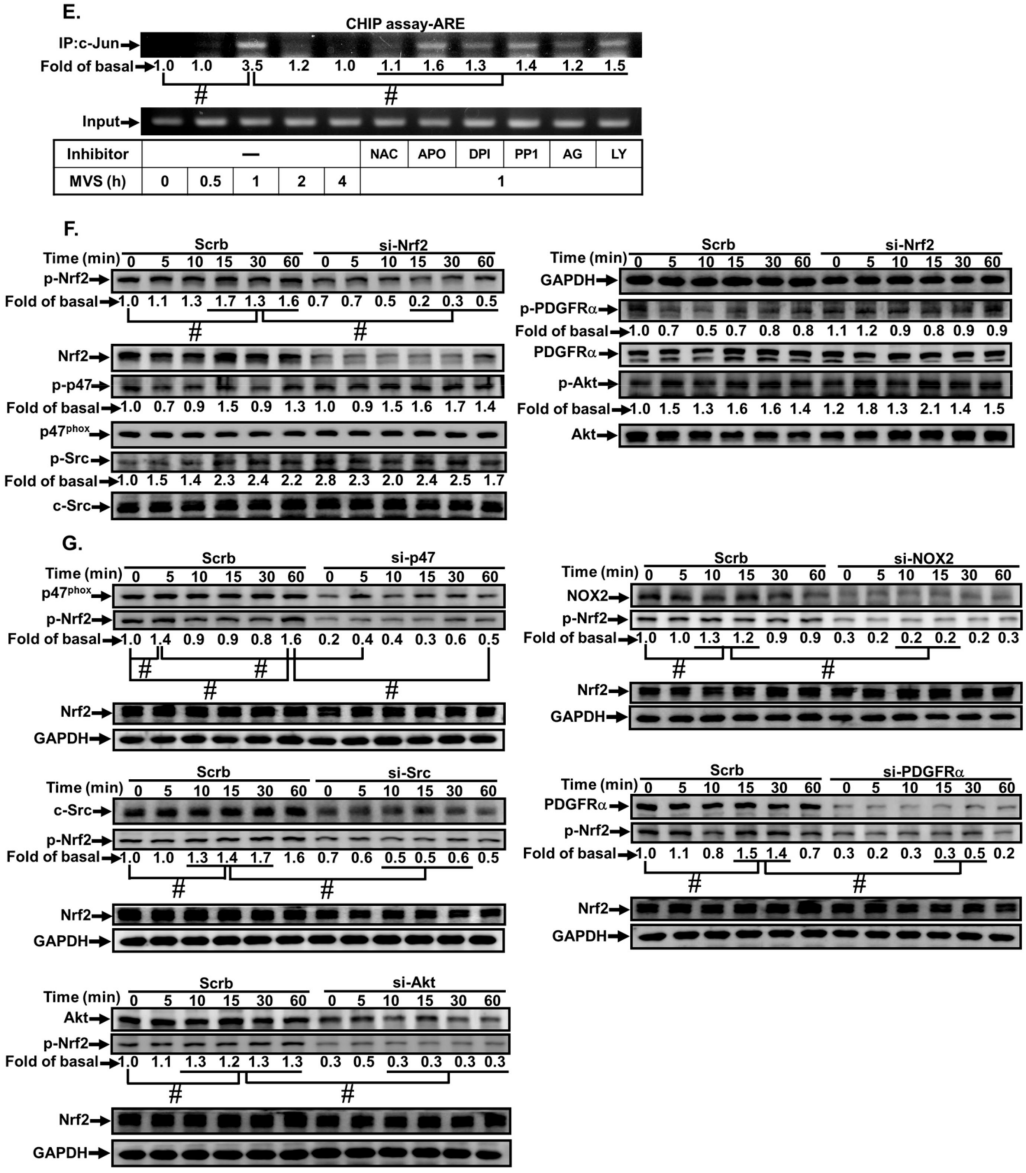
The involvement of the NOX/ROS/c-Src/PDGFRα/PI3K/Akt/Nrf2 cascade in the MVS-stimulated ARE promoter activity. (**A**) HPAEpiCs were transfected with Scrb or Nrf2 siRNA, and then incubated with MVS for 24 h. The levels of Nrf2, HO-1, and β-actin proteins were determined by Western blot. (**B**) Cells were pretreated with MVS (30 μM) for the indicated time intervals. The cytosol (CE) and nuclear (NE) fractions were prepared and analyzed by Western blot using anti-Nrf2, anti-GAPDH, or anti-Lamin A antibody. (**C**) HPAEpiCs were incubated with MVS (30 μM) for the indicated time intervals. Nuclear translocation of Nrf2 was determined by immunofluorescence staining. (**D**) HPAEpiCs transfected with ARE-luc plasmid were pretreated with PP1, AG1296, or LY294002 for 1 h, and then incubated with vehicle or MVS (30 μM) for 6 h. ARE promoter activity was determined. (**E**) Cells were pretreated without or with NAC, APO, DPI, PP1, AG1296, and LY294002 for 1 h, and then stimulated by 30 μM MVS for the indicated time intervals. The levels of Nrf2 binding to the ARE region of the HO-1 promoter were detected by a ChIP assay. HPAEpiCs were transfected with (**F**) Nrf2 or (**G**) p47*^phox^*, Nox2, c-Src, PDGFRα, and Akt siRNA, and then incubated with vehicle or 30 μM MVS for the indicated time intervals. Western blot was performed using anti-phospho-Nrf2, anti-Nrf2, anti-phospho-p47*^phox^*, anti-p47*^phox^*, anti-phospho-c-Src, anti-c-Src, anti-phospho-PDGFRα, anti-PDGFRα, anti-phospho-Akt, anti-Akt, or anti-GAPDH antibody. Data are expressed as mean ± SEM from five independent experiments (*n* = 5). ^#^*P* < 0.01 compared with the respective significantly different values as indicated.

Nrf2, an ROS-sensitive factor, plays a key role in regulating the physiological functions and maintaining cellular redox homeostasis through activation of ARE [31]. Nrf2 is a powerful modulator of genes including HO-1 expression, which protects against inflammation and apoptosis [14]. Nrf2 involvement in the MVS-induced HO-1 expression was confirmed by transfection with Nrf2 siRNA knocking down Nrf2 protein and attenuating the HO-1 level induced by MVS (Figure 8A). Phosphorylation of Nrf2 leads to its nuclear accumulation, which further binds to ARE on the HO-1 promoter. We found that MVS time-dependently stimulated Nrf2 translocation from cytosolic to nuclear fractions within 0.5–2 h, and then slightly declined (Figure 8B). We also noticed that the level of Nrf2 was increased within 4–6 h after treatment with MVS. The nuclear translocation of Nrf2 was further supported by the data of fluorescence images obtained by a fluorescence microscopy (Figure 8C). 

We further investigated whether MVS-stimulated transcriptional activation of ARE, an anti-oxidant-related promoter, occurred through binding with the transcription factor Nrf2 and turned on HO-1 gene expression. Cells were co-transfected with ARE-luc (ARE luciferase reporter construct) and pGal-luc (an internal control), and then stimulated by MVS. We found that MVS significantly stimulated ARE-luc reporter activity within six hours, which was reduced by pretreatment with the inhibitor of c-Src (PP1), PDGFRα (AG1296), or PI3K/Akt (LY294002) (Figure 8D). 

The role of Nrf2 and ARE interaction on HO-1 expression induced by MVS was evaluated using a ChIP assay with an anti-Nrf2 antibody. Pretreatment with NAC, APO, DPI, PP1, AG1296, or LY294002 diminished the MVS-stimulated DNA binding abilities of ARE on the HO-1 promoter (Figure 8E). Transfection with Nrf2 siRNA knocked down the level of Nrf2 protein and only reduced phosphorylation of Nrf2, but not p47*^phox^*, c-Src, PDGFRα, or Akt (Figure 8F), implying that Nrf2 is a downstream component of p47*^phox^*/Nox2/ROS/c-Src/PDGFRα/PI3K/Akt. We further examined whether MVS-induced Nrf2 activation was mediated through a p47*^phox^*/Nox2/ROS/c-Src/PDGFRα/PI3K/Akt pathway. MVS time-dependently stimulated Nrf2 phosphorylation, which was attenuated by transfection with p47*^phox^*, Nox2, c-Src, PDGFRα, or Akt siRNA (Figure 8G). These results suggested that MVS-induced HO-1 expression is mediated through a p47^phox^/Nox2/ROS/c-Src/PDGFRα/PI3K/Akt pathway, which then activates the Nrf2-ARE axis in HPAEpiCs.

## 4. Discussion

**Figure 9:**
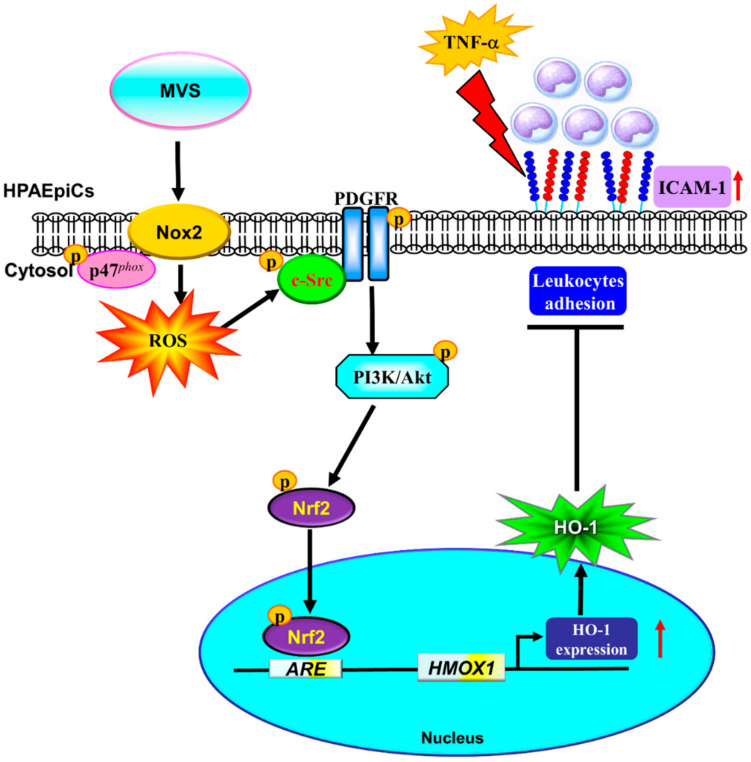
A schematic pathway for MVS-induced HO-1 expression in HPAEpiCs. MVS attenuated TNF-α-induced ICAM-1 expression and lung monocyte/leukocyte accumulation through upregulation of HO-1 via enhanced p47*^phox^*/Nox2 activity, resulting in the accumulation of intracellular ROS. Imbalance in oxidative stress promoted the phosphorylation of c-Src/PDGFRα/PI3K/Akt and then activation of Nrf2. Nuclear translocation of Nrf2 bound to the ARE region of the HO-1 promoter and increased the HO-1 gene expression in HPAEpiCs.

MVS has been widely used for the treatment of hyperlipidemia and exerts pleiotropic effects, including anti-inflammatory and anti-oxidant responses, which could attenuate the development of pulmonary diseases [8,9]. Our previous studies indicated that MVS attenuates S1P-induced airway inflammatory responses [10]. The detailed mechanisms of MVS protection against lung inflammatory responses have not been completely defined in HPAEpiCs. Here, we observed that pretreatment with MVS attenuated the TNF-α-induced expression of adhesion molecules via induction of anti-oxidant enzyme HO-1 in both in vitro and in vivo studies. The application of selective pharmacological inhibitors and siRNAs of p47*^phox^*, Nox2, c-Src, PDGFRα, Akt, and Nrf2 gene transcription revealed that these signaling components and transcription factors are involved in MVS-induced HO-1 expression. These results demonstrated that MVS-induced HO-1 expression is, at least in part, mediated through a p47*^phox^*/Nox2/ROS-generation-dependent activation of c-Src/PDGFRα/PI3K/Akt and then activates Nrf2/AREs pathway, which could protect against the pulmonary inflammatory responses challenged by TNF-α (Figure 9). 

Upregulation of HO-1 has beneficial effects in protecting against tissue oxidative stress and immune-mediated inflammation [16,37]. HO-1 gene (*HMOX1*) transcription and protein expression are induced by various stimuli and oxidative stress through activation of different transcription factors [11]. The molecular mechanisms of cytoprotective effects of HO-1 could down-regulate the responses of inflammatory mediators (e.g., LPS, TNF-α, or interleukins) via suppressing NF-κB-dependent pathways, apoptosis, and cell death [38,39]. However, the detailed mechanisms underlying MVS-induced HO-1 expression and protection against the TNF-α-mediated pulmonary inflammatory responses are not completely understood. In this study, up-regulation of HO-1 by MVS attenuated the TNF-α-induced ICAM-1 expression in both the mice model and HPAEpiCs. In contrast, deletion of *Hmox1* (*Hmox1*^-/-^) or inhibition of HO-1 activity exacerbated the severity of inflammatory diseases and resulted in early lethality or high incidence of mortality [11,14]. Our previous reports also indicated that up-regulation of HO-1 attenuates the expression of adhesion molecules, which is revered by an HO-1 activity inhibitor (ZnPP IX) or transfection with HO-1 siRNA in various types of cells and in vivo studies [33,40,41]. Here, we observed that the plasma concentrations of statins range from nM to μM levels under various conditions. However, to determine the effect of MVS on HO-1 expression, we used a higher concentration of MVS (30 μM). In this study, we could not exclude the alternative effects of MVS on these responses, which may be an important issue to consider in future study. Taken together, our results highlight that up-regulation of HO-1 by various reagents could help prevent respiratory inflammation. 

Low levels of ROS play a protective role in suppressing the inflammatory responses via up-regulation of anti-oxidant enzymes, including HO-1, through different intracellular signaling components [14]. Among them, p47*^phox^*/Nox2- or Nox4-dependent ROS production have been shown to suppress the inflammatory signaling pathways and ameliorate the inflammation during aging [42]. Thus, the shift in the balance between Nox/ROS production and anti-oxidants is extremely important in pathogenesis, which might act as a second messenger to regulate cellular functions and protect cells against injury [28]. Thus, the relationship between p47*^phox^*/Nox-dependent ROS generation and HO-1 expression was differentiated in HPAEpiCs. In line with these reports, our results showed that MVS-induced phosphorylation of p47*^phox^*, NADPH oxidase activity, ROS production, and HO-1 expression was blocked by the inhibitor of p47*^phox^* (APO) and Nox inhibitor (DPI), and ROS scavenger (NAC), or gene silencing through transfection with p47^phox^ and Nox2 siRNA, suggesting that p47^phox^/Nox/ROS plays a key role in the MVS-induced HO-1 pathway. These results are consistent with previous reports showing that p47^phox^/Nox-derived ROS generation is involved in HO-1 expression in HPAEpiCs [19,41]. 

Src family kinases might specifically regulate PDGFR signaling components. Activation of c-Src-associated with PDGFRα is involved in HO-1 expression in human tracheal smooth muscle cells [43,44]. Recently, we found that activation of the c-Src/PI3K/Akt or PDGFR/PI3K/Akt cascade is implicated in the ROS-dependent HO-1 expression, which protects against inflammatory responses [19,36,41]. We further differentiated the relationship between c-Src/PDGFRα/PI3K/Akt and HO-1 expression in HPAEpiCs challenged with MVS. Our results indicated that MVS-induced HO-1 expression was mediated through c-Src, PDGFRα, and Akt, which was attenuated by pretreatment with selective pharmacological inhibitors and transfection with respective siRNAs. Here, we also demonstrated that c-Src, PDGFRα, and PI3K/Akt are downstream components of Nox/ROS in MVS-mediated responses, since MVS-stimulated phosphorylation of c-Src/PDGFRα/Akt was attenuated by p47*^phox^* and Nox2 siRNA. The relationship among c-Src, PDGFRα, and PI3K/Akt in MVS-induced responses was further differentiated using the respective siRNAs. Phosphorylation of c-Src, PDGFRα, and Akt stimulated by MVS was reduced by transfection with c-Src siRNA, but no effect was observed on p47*^phox^* phosphorylation, suggesting that p47*^phox^* and Nox are upstream components of c-Src. The immunoprecipitation (IP) assay suggested that MVS stimulates the interaction between c-Src and PDGFRα to form a complex, leading to HO-1 expression in HPAEpiCs. Knockdown of PDFGRα expression attenuated phosphorylation of PDGFRα and Akt, but not c-Src and p47*^phox^*, suggesting that PDGFRα, PI3K, and Akt are downstream components of p47*^phox^*/c-Src stimulated by MVS. Taken together, these results verify that the mechanisms underlying MVS-induced HO-1 expression are mediated through a p47*^phox^*/Nox/ROS-dependent activation of the c-Src/PDGFRα/PI3K/Akt cascade in HPAEpiCs. 

Multiple signaling pathways and transcription factors play important roles in regulation of several cellular functions [45]. ROS generation acts as a second messenger that triggers keap1 proteolysis and thereby activates Nrf2 signaling [28]. The transcription factor keap1-Nrf2 system interacts with the AREs, leading to the transcriptional activation of anti-oxidant enzymes such as HO-1 [21,32,46]. In the normal state, the cytosolic repressor protein keap1 binds to Nrf2, keeps Nrf2 in the cytoplasm, and promotes its degradation [47]. In contrast, exposure to oxidative stress modifies cysteine residues on keap1, leading to conformational changes, thereby releasing Nrf2 and translocates into nucleus binding to ARE-driven expression of HO-1. The up-regulation of HO-1 may be a potential therapeutic intervention for management of pulmonary inflammatory diseases [32,48]. We also noted that activated Nrf2 could regulate the expression of several anti-oxidant proteins, such as NADPH quinone dehydrogenase 1 and glutamate cysteine ligase catalytic subunit. Thus, we cannot exclude the roles of NADPH quinone dehydrogenase 1 and glutamate cysteine ligase catalytic subunit on the inhibitory effects of MVS on ICAM-1 expression triggered by TNF-α. The roles of the other anti-oxidant proteins induced by statins may be worthy of further investigation.

The roles of transcriptional factors involved in the MVS-induced HO-1 expression mediated through various signaling components were investigated in HPAEpiC. Our results indicated that Nrf2 plays a key role in HO-1 expression induced by MVS. In this study, MVS-stimulated Nrf2 phosphorylation and nuclear accumulation enhanced the recruitment of Nrf2/ARE to HO-1 promoter, which was attenuated by pretreatment with NAC, APO, DPI, PP1, AG1296, and LY294002, or transfection with their respective siRNAs. These results suggest that MVS-induced HO-1 expression is mediated through activation of Nrf2 regulated by activation of the p47*^phox^*/Nox2/ROS/c-Src/PDGFRα/PI3K/Akt cascade in HPAEpiCs. These results are consistent with the studies that reported that activated Nrf2 plays an important role in HO-1 expression in various types of cells [14,49]. However, other transcription factors, such as AP-1 and Sp1, may also be involved in the up-regulation of HO-1 in responses to other activators [50]. Therefore, the roles of AP-1 and Sp1 implicated in the MVS-induced HO-1 expression are important issues that require future study.

## 5. Conclusions

We found that MVS attenuated TNF-α-induced ICAM-1 expression, at least in part mediated through p47*^phox^*/Nox/ROS/c-Src/PDGFRα/PI3K/Akt/Nrf2/ARE/HO-1, which protected against inflammatory lung diseases. Although several mechanisms elucidate anti-hyperlipidemic effects of statins in clinics, these findings expand the potential application of statins as intervention for the prevention or treatment of pulmonary inflammatory diseases. Better understanding of the mechanisms of MVS-induced HO-1 expression might provide a novel application of statins for preventive or therapeutic treatment and management of inflammatory pulmonary disease.
