# Correction: Lin et al. Induction of HO-1 by Mevastatin Mediated via a Nox/ROS-Dependent c-Src/PDGFRα/PI3K/Akt/Nrf2/ARE Cascade Suppresses TNF-α-Induced Lung Inflammation. *J. Clin. Med.* 2020, *9*, 226

**DOI:** 10.3390/jcm14155390

**Published:** 2025-07-31

**Authors:** Chih-Chung Lin, Wei-Ning Lin, Rou-Ling Cho, Chien-Chung Yang, Yi-Cheng Yeh, Li-Der Hsiao, Hui-Ching Tseng, Chuen-Mao Yang

**Affiliations:** 1 Department of Anesthetics, Chang Gung Memorial Hospital at Linkuo, Kwei-San, Tao-Yuan 33302, Taiwan; chihchung@adm.cgmh.org.tw; 2 Graduate Institution of Biomedical and Pharmaceutical Science, College of Medicine, Fu Jen Catholic University, New Taipei City 24205, Taiwan; 081551@mail.fju.edu.tw; 3 Department of Pharmacology, College of Medicine, China Medical University, Taichung 40402, Taiwan; royeariel760918@gmail.com (R.-L.C.); xmasmilk@hotmail.com (Y.-C.Y.); lidesiao@livemail.tw (L.-D.H.); huiching1205@yahoo.com.tw (H.-C.T.); 4 Department of Traditional Chinese Medicine, Chang Gung Memorial Hospital at Tao-Yuan, Kwei-San, Tao-Yuan 33302, Taiwan; r55161@cgmh.org.tw; 5 School of Traditional Chinese Medicine, College of Medicine, Chang Gung University, Kwei-San, Tao-Yuan 33302, Taiwan; 6 Department of Post-Baccalaureate Veterinary Medicine, College of Medical and Health Science, Asia University, Wufeng, Taichung 41354, Taiwan

## Error in Figure

In the original publication [1], there was a mistake in Figure 3A as published. We found some errors in Figure 3A during the composition of internal control beta-actin. We have carefully repeated these experiments, analyzed them, and provided the exact results shown in the attached file below. The corrected Figure 3A appears below. The authors state that the scientific conclusions are unaffected. This correction was approved by the Academic Editor. The original publication has also been updated.

## Figures and Tables

**Figure 3:**
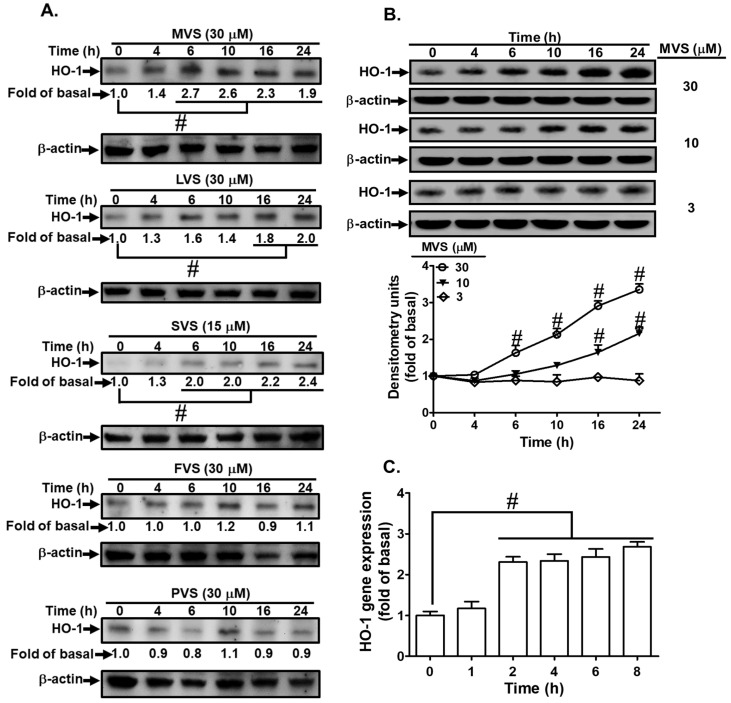
Statins induce HO-1 expression in HPAEpiCs. (**A**) The effects of statins on HO-1 expression; the cells were incubated with MVS, lovastatin (LVS), simvastatin (SVS), fluvastatin (FVS), and pravastatin (PVS) for the indicated time intervals. (**B**) HPAEpiCs were incubated with different concentrations of MVS (3, 10, and 30 μM) for the indicated time intervals. (**A**,**B**) The levels of HO-1 and β-actin protein expression were determined by Western blot. (**C**) Total RNA was isolated from HPAEpiCs treated with MVS (30 μM) for the indicated time intervals. The levels of HO-1 mRNA were determined by real-time PCR. Data are expressed as mean ± SEM from five independent experiments (*n* = 5). ^#^ *p* < 0.01 compared with the cells exposed to vehicle (0 h) alone.
